# Biomimetic Targeted Theranostic Nanoparticles for Breast Cancer Treatment

**DOI:** 10.3390/molecules27196473

**Published:** 2022-10-01

**Authors:** Suphalak Khamruang Marshall, Pavimol Angsantikul, Zhiqing Pang, Norased Nasongkla, Rusnah Syahila Duali Hussen, Soracha D. Thamphiwatana

**Affiliations:** 1 Department of Biomedical Sciences and Biomedical Engineering, Faculty of Medicine, Prince of Songkla University, Songkhla 90110, Thailand; 2 Department of Radiology, Faculty of Medicine, Prince of Songkla University, Songkhla 90110, Thailand; 3 Center for Biomedical Research, Population Council, 1230 York Ave, New York, NY 10065, USA; 4 Key Laboratory of Smart Drug Delivery, Ministry of Education, School of Pharmacy, Fudan University, Shanghai 201203, China; 5 Department of Biomedical Engineering, Faculty of Engineering, Mahidol University, Nakorn Pathom 73170, Thailand; 6 Chemistry Department, Faculty of Science, University of Malaya, Kuala Lumpur 50603, Malaysia

**Keywords:** nanomedicine, biomimetic, nanoparticles, theranostics, cancer

## Abstract

The development of biomimetic drug delivery systems for biomedical applications has attracted significant research attention. As the use of cell membrane as a surface coating has shown to be a promising platform for several disease treatments. Cell-membrane-coated nanoparticles exhibit enhanced immunocompatibility and prolonged circulation time. Herein, human red blood cell (RBC) membrane-cloaked nanoparticles with enhanced targeting functionality were designed as a targeted nanotheranostic against cancer. Naturally, derived human RBC membrane modified with targeting ligands coated onto polymeric nanoparticle cores containing both chemotherapy and imaging agent. Using epithelial cell adhesion molecule (EpCAM)-positive MCF-7 breast cancer cells as a disease model, the nature-inspired targeted theranostic human red blood cell membrane-coated polymeric nanoparticles (TT-RBC-NPs) platform was capable of not only specifically binding to targeted cancer cells, effectively delivering doxorubicin (DOX), but also visualizing the targeted cancer cells. The TT-RBC-NPs achieved an extended-release profile, with the majority of the drug release occurring within 5 days. The TT-RBC-NPs enabled enhanced cytotoxic efficacy against EpCAM positive MCF-7 breast cancer over the non-targeted NPs. Additionally, fluorescence images of the targeted cancer cells incubated with the TT-RBC-NPs visually indicated the increased cellular uptake of TT-RBC-NPs inside the breast cancer cells. Taken together, this TT-RBC-NP platform sets the foundation for the next-generation stealth theranostic platforms for systemic cargo delivery for treatment and diagnostic of cancer.

## 1. Introduction

Cancer is the second leading cause of death globally. In 2022, there will be approximately 4.8 million and 2.4 million new cancer cases and 3.2 million and 0.64 million cancer deaths in China and the United States of America [1], respectively. The most common cancer in the USA is breast cancer [2]. Breast cancer was the highest-ranking cause of new cases at 2.26 million, representing 11.7% of new cases and 684,996 deaths. Between 2020 and 2040, cancer cases are predicted to rise by 47% to 28.4 million cases [3]. Currently the major treatment options are surgery, radiation, and chemotherapy, although these treatments have limitations and adverse side effects [4]. However, surgery cannot be offered to patients in whom the cancer has spread, with only the option of chemotherapy treatment offered. Chemotherapy drugs, which kill the rapidly dividing cancer cells, have been the mainstay of cancer treatment. However, they have substantial harmful side effects on the patient’s immune system [5]. Most chemotherapeutic drugs have a short half-life and a high clearance rate in the bloodstream. Today, the vast majority of clinically used drugs are low-molecular-weight compounds that distribute evenly in the body and diffuse quickly into healthy tissues. As a result, the target site receives only small amounts of the drugs, such as doxorubicin (DOX), giving them a narrow therapeutic index with a sensitive balance between efficacy and toxicity [6]. Various studies have shown that frequent low-dose administration proves to be more effective than less-frequent high-dose administration, suggesting that a low but steady in vivo drug dosage can possibly enhance the drugs treatment profile [7]. To extend the circulation half-life of anthracycline drugs, liposomal formulations such as Doxil^®^, DaunoXome^®^, and Myocet^®^ have been developed and have shown varying degrees of success in many disease treatments clinically [8].

Recently, cancer treatment has become more tailored to the specific type of cancer, with many advances being made in drug delivery systems. Conventional therapeutic agents suffer from inappropriate pharmacokinetics, solubility, and biodistribution as well as limited selectivity and a lack of effectiveness [9,10]. These side effects that limit the maximum allowable systemic dose result in insufficient drug concentrations reaching tumor sites. Nanotheranostics take advantage of nanoscale drug delivery platforms to address the physiological barriers in many disease treatments. Nanoscale drug delivery systems are based on a number of key advantages: (a) improved solubility of hydrophobic compounds, (b) protection of molecules from unwanted interactions with biological components and improve stability, (c) enabling of controlled release of the payload, and (d) favorably changes the pharmacokinetics and biodistribution.

An effective nanoscale drug delivery system design needs to overcome human biological barriers and factors such as nanomaterial, size, shape, hydrophobicity, and surface chemistry, to name a few that need consideration [11]. More recently, the development of nanotheranostics for cancer treatment, which is a combination of both therapeutics and diagnostics, has been verified as successfully delivering cancer drug, providing imaging-guided focal therapy, and also monitoring patients’ post-treatment response [12].

For drug delivery nanocarriers, overcoming opsonization and sequestration by the mononuclear phagocyte system (MPS) in the lymph nodes, liver, and spleen is a key requirement [13]. Consequently, polyethylene glycol (PEG) became the gold standard for stealth coating NPs to evade the mononuclear phagocyte system. However, anti-PEG immunological response observations have generated additional research on the biological consequences of anti-PEG antibodies [14]. This has triggered researchers’ interest in searching for better alternatives to PEG coating. Long-circulation strategies inspired from biological materials such as red blood cells (RBCs) membrane, macrophage membrane or platelet membrane has become very attractive [15,16,17,18,19,20]. These biomimetic strategies have shown to possess MPS evasion capability as they are able to camouflage nanocarriers and bypass immune surveillance.

In contrast to the conventional approach of covalently conjugating functional proteins to nanoparticles, this model translocates the entire cellular membrane to the nanoparticle’s surface and provides the most natural medium for anchoring membrane proteins and exposing their active domains on the surface of the nanoparticles. [15,17,21,22,23,24]. This creative engineering can effectively avoid common issues associated with chemical modifications such as protein denaturing, particle dimerization, and blockage of the active sites [25,26,27,28], Moreover, this top-down approach bypasses labor-intensive and costly surface protein identification, purification, mass production, and conjugation.

Despite several advantages of biomimetic nanoparticles, some of their drawbacks is lack of selectivity and specificity, which can be overcome by improving the targeting accuracy. This can be achieved by conjugating targeting ligands on the external surface of NPs. These molecules have the ability to distinguish specific ligands that are expressed by the targeted cancer cells [29]. For instance, Luk et al. and his team investigated the efficacy of mouse RBC coated DOX-loaded nanoparticles (RBC-NP) against cancer using a murine lymphoma model [16]. The results were promising; however, their process relied only on passive targeting or enhanced permeability and retention (EPR) effect. The development of biomimetic drug delivery systems for biomedical applications has received significant research attention. Therefore, active targeting would potentially improve therapeutic efficacy.

**Figure 1:**
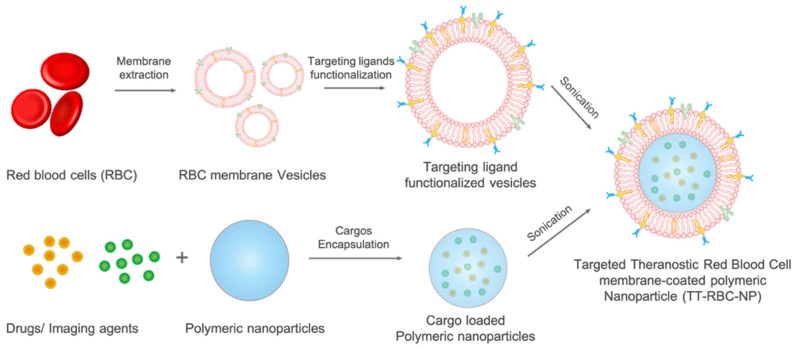
Schematic illustration of the targeted theranostic red blood cell membrane-coated nanoparticles (TT-RBC-NPs). Red blood cell membrane is extracted by hypotonic treatment, sonication, and extrusion. Targeting ligands are functionalized by lipid insertion into red blood cells membrane vesicles. The targeting ligand functionalized vesicles are coated on cargo-loaded polymeric cores to form TT-RBC-NPs.

Herein, our study further developed biomimetic NPs by incorporating active targeting and an imaging agent as biomimetic targeted theranostic nanoparticles. The targeting functionality was added on naturally derived biomimetic nanoparticles. CD326 (the cell surface protein epithelial cell adhesion molecule, EpCAM) was chosen as the targeting moiety because of its high expression level in various adenocarcinoma cell lines and low expression level in normal cells [30]. A unique, nature-inspired, long-circulating nanoparticles containing chemotherapy and imaging agents was developed to target and treat EpCAM+ breast cancer with minimal uptake to non-targeted cells as well as to visualize tumor; hence, treatment progress could be monitored at the same time (Figure 1).

## 2. Results and Discussion

A theranostic nanoparticle delivery system ultimately depends on its ability to diagnose cancer, provide a treatment tailored to the diagnostic, and finally be able to monitor the treatment efficacy. Inspired by the prolonged circulation and outstanding biocompatibility of RBC-membrane coated polymeric nanoparticles with a 39.6 hours half elimination compared to PEGylated nanoparticles 15.8 h half-live [17], the current study fabricated targeted theranostic human red blood cell membrane-coated nanoparticles (TT-RBC-NPs) and evaluated the targeted delivery of chemotherapeutic and imaging agents to MCF-7 breast cancer in a 2D and 3D cell culture model.

### 2.1. Synthesis and Characterization of TT-RBC-NPs

**Figure 2:**
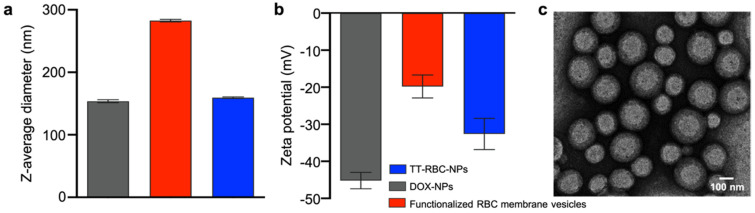
Physicochemical characterization of TT-RBC-NPs. (**a**) Hydrodynamic size and (**b**) zeta potential of non-coated doxorubicin-loaded nanoparticles (DOX NPs), RBC membrane vesicles, and TT-RBC NPs (mean ± SD, *n* = 3). (**c**) Transmission electron micrographs of TT-RBC-NPs. Scale bar = 100 nm.

DOX-loaded PLGA nanoparticles were prepared using the double emulsion solvent evaporation method. In addition, various strategies for attaching bioactive moieties to nanoparticles have been established in recent years. These interactions are categorized into the following approaches: adsorption, biotin-streptavidin conjugation, covalent coupling, ligand-receptor conjugation, and lipid fusion. In order to successfully amalgamate the human RBC membrane vesicle coating and the anti-EpCAM targeting ligand on the surface of the PLGA NP, lipid insertion method was utilized in this study to synthesize the surface functionalization of the anti-EpCAM on human RBC membrane. Functionalized human RBC membrane was then coated onto the polymeric cores using a sonication approach as previously described [16]. For every 1 mg of PLGA, 3 × 10^12^ molecules of biotinylated anti-EpCAM were introduced onto the surface of nanoparticles. Using Nanoparticle Tracking Analyzer [31] (NanoSight, Malvern Panalytical), approximately 9.11 × 10^10^ particles/1 mg PLGA was measured. Based on the NTA result, the number of anti-EpCAM molecules per particle was estimated to be ~33, which is consistent with previously reported works [32,33]. The physicochemical properties of the TT-RBC-NPs were analyzed with dynamic light scattering (DLS) using a Malvern Zetasizer. The particle size and zeta potential were investigated. Functionalized RBC membrane vesicles were approximately 292.6 nm in z-average hydrodynamic diameter. Coating of ~153.7 nm DOX-loaded PLGA cores with functionalized RBC membrane vesicles resulted in a size increase to approximately 159.4 nm (z-average size) (Figure 2a). Additionally, the TT-RBC-NPs’ surface charge changed by ~12 mV, increasing from ~-45 mV to ~-33 mV (Figure 2b). This has been reported in previous findings, as the membrane layer adds to the hydrodynamic size and the less negatively charged RBC membrane coating blocks out the carboxyl groups on the core surface which have a high negative charge [34]. NPs with a zeta potential greater than or equal to +30 mV or less than −30 mV have sufficient repulsive force for enhanced particle size and colloidal physical stability [35]. In addition, Elvin Blanco et al. also observed that the zeta potential of a nanoparticle is a feature that allows it to pass through the cell membrane, and negatively charged NPs inhibit excretion by the kidney and reticuloendothelial system (RES) [36]. The core-shell structure of the drug-loaded TT-RBC-NPs was confirmed by visualization using transmission electron microscopy with uranyl acetate negative staining (Figure 2c). The physicochemical properties of nanocarriers play a significant role in nanomedicine. Both the nanoparticle size, and surface functionalization have a major impact on the circulation half-life after an intravenous administration [37]. Many studies reported that nanoparticles in the size range of 100–200 nm have the best effects for theranostic applications [38]. In addition, the drug administration effectiveness of nanocarriers is determined by the nanoparticle diameter, PDI, and zeta potential. Nanoparticles with diameters ranging from 100 nm to 2000 nm are administered intravenously or intramuscularly [39].

### 2.2. Optimization and Stability of TT-RBC-NPs

**Figure 3:**
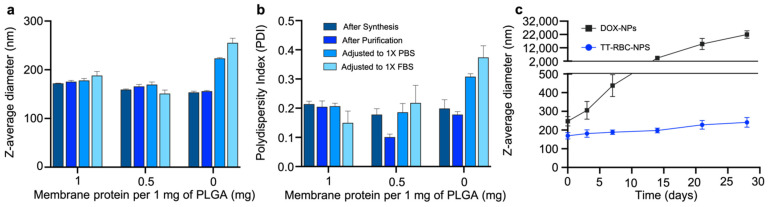
Optimization and stability of TT-RBC-NPs. (**a**) Z-average size and **b**) Polydispersity index (PDI) as measured by dynamic light scattering (DLS) at varying RBC membrane protein to PLGA weight ratios of doxorubicin-loaded NPs immediately after synthesis, after purification, after adjusting to 1× PBS, and after adjusting to 1× FBS. (**c**) Stability of TT-RBC NPs made at a membrane to core ratio of 0.5 mg protein per 1 mg PLGA versus DOX-NPs over time. Data given as mean ± SD (*n* = 3).

Modified RBC membrane coatings were optimized by synthesizing at membrane-to-core weight ratios at 0.5 and 1 mg of membrane protein per 1 mg of PLGA particles (Figure 3a). When the particles were adjusted to 1× phosphate buffered saline (PBS), and 1× fetal bovine serum (FBS), a significant increase in the z-average diameter of the non-coated nanoparticles was observed. While the hydrodynamic size of the coated nanoparticles, at membrane-to-core weight ratios at both 0.5 and 1 mg of membrane protein per 1 mg of PLGA particles remained unchanged. This suggested complete coverage, with the PLGA core surfaces not exposed to charge screening, resulting in good stability in ionic buffers. Furthermore, the polydispersity index (PDI) of particles at both 0.5 and 1 membrane-to-core weight ratios were below ~0.2 and considered as monodisperse particles with narrow particle size distribution (Figure 3b). Therefore, the lower membrane-to-core ratio, 0.5 mg of membrane protein per 1 mg of PLGA particles was chosen for subsequent studies. At this ratio, the TT-RBC-NPs maintained size stability with minimal size increase throughout 1 month of storage at 4 °C (Figure 3c). On the contrary, DOX-NPs size increased over the time of storage and aggregation was observed.

### 2.3. Drug Loading and Cumulative Release of TT-RBC-NPs

**Figure 4:**
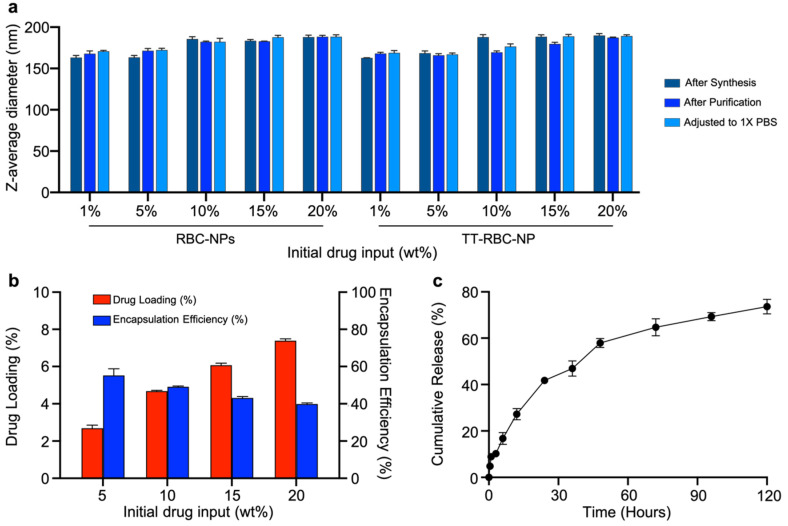
Drug loading and cumulative release of doxorubicin from TT-RBC-NPs: (**a**) Z-average size of RBC-NPs and TT-RBC-NPs right after synthesis, after purification, after adjusting to 1× PBS at various initial drug input. (**b**) Drug loading yield and encapsulation efficiency (%) of DOX in TT-RBC-NPs at varying initial drug inputs. (**c**) Cumulative release profile (%) of DOX from TT-RBC-NPs with 6 wt% of DOX loading yield over a period of 5 days. Results represent mean ± SD (*n* = 3).

Drug loading into the polymeric cores could be controlled by varying the initial drug input. The initial input concentrations of DOX were varied from 1–20wt% (DOX weight/PLGA weight). The sizes of unmodified RBC membrane coated DOX-loaded NPs, herein denoted as RBC-NPs and TT-RBC-NPs with various initial drug inputs were measured (Figure 4a). Comparable sizes of all samples were observed with a slight increase in size as initial input increased. Moreover, they were all stable when the particles were transferred to 1× PBS, suggesting complete membrane coverage. The drug-loading yield and encapsulation efficacy of the TT-RBC-NPs were investigated to find the optimal formulation. Figure 4b demonstrates that an increase in the initial drug input of DOX into the PLGA core, resulted in an increase in the drug loading yield of DOX while the encapsulation efficacy decreased. This is consistent with a previously reported study [16]. The optimal formulation selected was 15 wt% DOX input, corresponding to approximately 6 wt% loading with an encapsulation efficacy ~45%. This formulation was used for subsequent experiments. The cumulative drug release study of the optimal formulation of the TT-RBC-NPs was investigated over 5 days. The cumulative release profile demonstrated that DOX sustained release from the TT-RBC-NPs over time. Approximately 50% of DOX released within the first 48 h, and approximately 80% of DOX released over the course of 5 days. These results indicate that the majority of the release occurs within the first 72 h. The prolonged DOX release is due to the slow diffusion of drug molecules through the matrix of PLGA NPs as well as the RBC membrane coating as another diffusional layer. Plotting the release profile against the square root of time yielded linear fitting with R^2^ = 0.98. This good fitting of Higuchi kinetic release model implies a diffusion-sustained drug release mechanism (Figure S1). This phenomenon has been previously reported [40,41,42].

### 2.4. In Vitro 2D Therapeutic Efficacy

**Figure 5:**
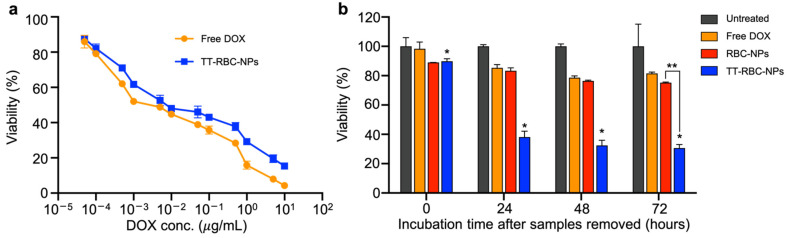
In vitro therapeutic response of TT-RBC-NPs. (**a**) In vitro cytotoxicity of free DOX and TT-RBC-NPs against MCF-7 breast cancer cells after 72 h incubation. (**b**) In vitro efficacy of free DOX, RBC-NPs and TT-RBC-NP (equivalent of 1 µg/mL DOX) against MCF-7 cells. In this assay, samples were incubated for 2 h, and cells were subsequently washed and incubated in fresh medium for additional 0, 24, 48 and 72 h before quantifying toxicity. Data represents mean ± SD (*n* = 3). * the significance between TT-RBC-NPs at 0, 24, 48, and 72 h incubation (*p* < 0.05). ** the significance between RBC-NPs and TT-RBC-NPs at 72 h incubation (*p* < 0.05).

To determine the in vitro therapeutic efficacy, an MTS assay cytotoxicity test was used to assess the cytotoxicity effects of the TT-RBC-NPs and free DOX at various concentrations. The cytotoxicity effect was evaluated on MCF-7 breast cancer cells (EpCAM+) after 72 h of incubation. The results suggested that free DOX had marginally better toxicity on the MCF-7 cells in comparison with the TT-RBC-NPs. The half maximal inhibitory concentration (IC_50_) of free DOX against MCF-7 was 1 ng/mL while the IC_50_ of the TT-RBC-NPs was 10 ng/mL, as shown in Figure 5a. Non-drug loaded nanoparticles showed no cytotoxicity against targeted cells (Figure S2). In short, the release of DOX from the TT-RBC-NPs was incomplete after 72 h incubation. Moreover, free DOX was able to more readily diffuse into the individual cancer cells in 2D cultures over a long incubation period. However, it was previously reported that binding ability of targeted nanoparticles was within 2 h [43]. Therefore, we prepared TT-RBC-NPs, RBC-NPs (non-targeted RBC coated DOX-loaded NPs), and free DOX (equivalent of 1 µg/mL DOX) to evaluate their relative cytotoxicity against MCF-7 breast cancer cells using in vitro cell viability assays (Figure 5b). In this study, we incubated various samples with MCF-7 cells for 2 h, washed cells with PBS to removed unbound, excess samples, and then supplemented them with fresh cell culture medium. Cells were then incubated for another 0, 24, 48 and 72 h before carrying out a viability assay. The results show that the TT-RBC-NPs have the highest cytotoxicity toward targeted MCF-7 (EpCAM+) cells. Cell viability was approximately 38, 32, and 30% for 24, 48 and 72 h incubation, respectively. Significantly less cytotoxicity of free DOX and RBC-NPs was observed. This is possibly because the unbound free DOX and RBC-NPs were washed out, while the TT-RBC-NPs bound to MCF-7 and were taken up via active mechanisms such as endocytosis [44].

### 2.5. In Vitro Cellular Toxicity of TT-RBC-NPs in Three-Dimensional (3D) Spheroids

**Figure 6:**
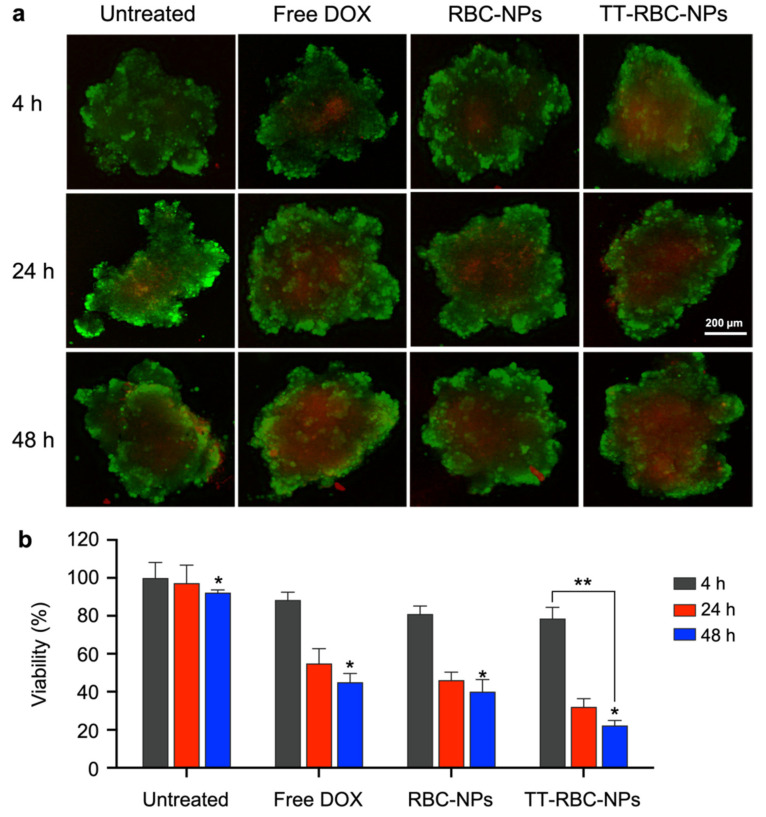
In vitro cellular toxicity of TT-RBC-NPs in three-dimensional (3D) spheroids. (**a**) In vitro live/dead cell imaging of tumor spheroid untreated, and treated with free DOX, RBC-NPs and TT-RBC-NPs (equivalent of 1 µg/mL DOX) for a period of 4, 24 and 48 h. Two-color fluorescence, live (green channel) and dead (red channel), enables evaluation of live and dead cells to determine cell viability. Scale bar = 200 µm. (**b**) In vitro cytotoxicity study using CellTiter-Glo^®^ 3D cell assay of untreated, free DOX, RBC-NPs and TT-RBC-NPs against 3D MCF-7 spheroids. Results represent mean ± SD (*n* = 3). * the significance between Untreated, Free DOX, RBC-NPs, and TT-RBC-NPs at 48 h incubation (*p* < 0.05). ** the significance between 4 h and 48 h incubation of TT-RBC-NPs (*p* < 0.05).

The TT-RBC-NPs were further evaluated on their therapeutic efficacy against 3D MCF-7 tumor spheroids using a live/dead assay. The spherical aggregates produced from MCF-7 cells were treated by free DOX, RBC-NPs, and TT-RBC-NPs at a fixed DOX concentration of 1 µg/mL for 4, 24, and 48 h. Live/dead cell assay images are represented in Figure 6a. Two-color fluorescence, green [45] and red (dead), enables the evaluation of live and dead cells in a 3D culture. The spheroid size in all groups was comparable but a difference between dead/live cells ratios were visualized. In addition, untreated spheroids were used as a control, indicating most cells inside spheroids are still alive. In contrast, spheroids treated with free DOX, RBC-NPs, and TT-RBC-NPs dead cells were observed (Figure 6a). The images revealed that the highest number of dead cells were visualized when treated with the TT-RBC-NPs for 48 h, resulting in approximately a two-fold higher fluorescence intensity. However, it was very difficult to distinguish overall efficacy using only fluorescence images due to the complexity of the 3D spheroid mass. Therefore, a quantitative cell viability assay was performed using the CellTiter-Glo^®^ 3D cell viability assay (Figure 6b). The viability percentage after 4 h incubation, reduce significantly for all samples. The results obtained in this assay aligned with comparable with 2D MTS assay. At 48 h incubation time, the TT-RBC-NP treatment significantly reduced cell viability (22 ± 2.5%) within the spheroid masses, in comparison to free DOX (45 ± 5%), and RBC-NPs (40 ± 6%). In addition, the TT-RBC-NPs treated spheroids showed the highest cytotoxic effect compared to the other treatment groups against 3D MCF-7 spheroids. Similarly, Lee et al. designed a targeted doxorubicin (DOX)-engineered PLGA nanoparticle with phloretin with the combination of sialic acid to enhance tumor targeting and penetration. They assessed the tumor penetration capability of the NPs in an A549 cell 3D spheroid model before they conducted in vivo studies. From their 3D model, it was surmised that the DOX PLGA NPs efficiently infiltrated into the heterogeneous tumor [46].

### 2.6. In Vitro Targeting Ability Study of TT-RBC-NPs

**Figure 7:**
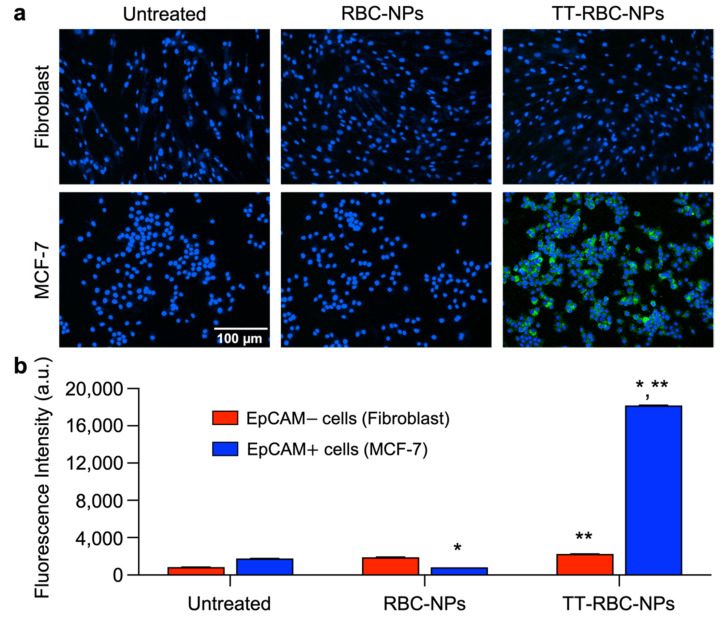
Targeting ability study of TT-RBC-NPs by fluorescence microscopy. (**a**) Fluorescence microscopy images of MCF-7 (EpCAM+) and skin fibroblast (EpCAM–) treated with RBC-NPs (non-targeted NPs) and TT-RBC-NPs (targeted NPs). All samples were labeled with FITC (green channel). After 1 h incubation, cells were washed and incubated for another 1 h in fresh medium before imaging. The nucleus was stained with DAPI (blue channel). Scale bar = 100 µm. (**b**) Quantification of mean fluorescence intensities of RBC-NPs and TT-RBC-NPs on MCF-7 (EpCAM+) and skin fibroblast (EpCAM–) cells. Bars represent mean ± SD (*n* = 3). * the significance between RBC-NPs of EpCAM+ cells (MCF-7) and TT-RBC-NPs of EpCAM+ cells (MCF-7) (*p* < 0.05). ** the significance between TT-RBC-NPs of EpCAM– cells (fibroblast) and TT-RBC-NPs of EpCAM+ cells (MCF-7) (*p* < 0.05).

The in vitro targeting capability of the TT-RBC-NPs against MCF-7 (targeted cells) and fibroblast (non-targeted cells) was observed under fluorescent microscopy (Figure 7a). This confirmed the nanotheranostic carrier targeting efficacy to breast cancer cells that overexpressed EpCAM on the cell surface. All samples were labeled with FITC as a model imaging agent. RBC-NPs, non-targeted DOX-NPs, used as a non-targeted control. After 1 h incubation, both MCF-7 (targeted cells) and fibroblast (non-targeted cells) were washed and incubated for another 1 h in fresh medium before imaging. The nucleus was stained with DAPI (blue channel), and the fluorescence images demonstrated the binding of the TT-RBC-NPs to the targeted cells, MCF-7 (EpCAM+). However, they did not bind to the non-targeted skin fibroblast (EpCAM−) cells, suggesting an efficient specificity of TT-RBC-NPs toward EpCAM+ cells. In contrast, RBC-NPs only barely bound to the cells after the same incubation duration. Moreover, the quantification of RBC-NP and TT-RBC-NP mean fluorescence intensities on MCF-7 (EpCAM+) and skin fibroblast (EpCAM−) cells (Figure 7b) confirmed similar results. A high FITC fluorescence intensity from the nanoparticles was detected on the MCF-7 cells when treated with the TT-RBC-NPs, with a 23-fold higher intensity than the RBC-NPs (non-targeted NPs). While minimal FITC fluorescence intensity was detected when treated with RBC-NPs non-targeted control group.

Furthermore, our results are consistent with previous studies that report that the binding ability of targeted NPs occurs within 1-2 h after incubation, and it suggested that internalization via a receptor-mediated endocytosis mechanism is likely to occur after efficient specific binding [43]. Additionally, the role of DSPE-PEG-biotin was to create a PEG shell to enhance the stability of the PLGA NPs by producing both steric hindrance and electrostatic repulsion [47]. In addition, our TT-RBC-NP platform contains biotin, a low-molecular-weight ligand that targets the sodium-dependent multivitamin transporter on tumor cells. Biotin-conjugated molecules can play a role to improve the cellular uptake [48]. Similarly, Huang, Mengyi, et al. designed biotin-glucose dual-targeting liposomes, and their results indicated the addition of biotin-enhanced targeting to MCF-7 cells [49].

**Figure 8:**
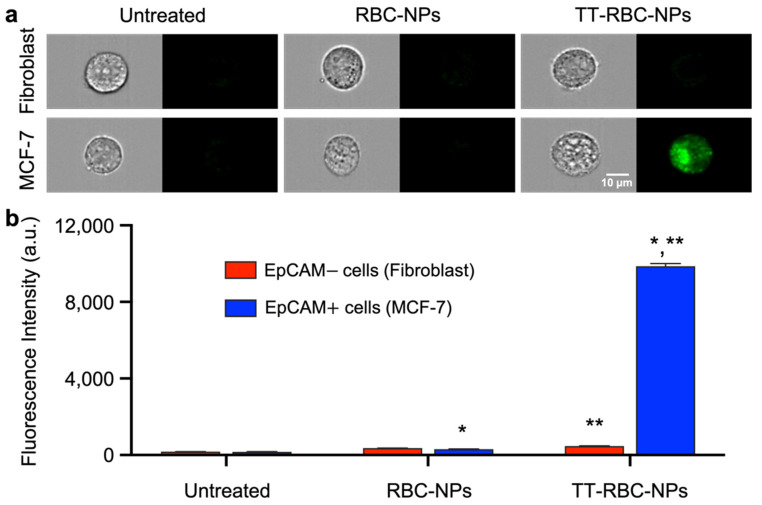
TT-RBC-NPs bind to MCF-7 (EpCAM+) cell. (**a**) Fluorescence images of MCF-7 (EpCAM+) and skin fibroblast (EpCAM–) cells treated with RBC-NPs (non-targeted NPs) and TT-RBC-NPs (targeted NPs). Images and fluorescent signals were measured by flow cytometer. The cancer cell morphology was imaged in bright field. All samples were labeled with FITC (green channel). Scale bar = 10 µm. (**b**) Quantification of mean fluorescence intensities of untreated, RBC-NPs and TT-RBC-NPs on skin fibroblast and MCF-7 cells. Bars represent mean ± SD (*n* = 3). * the significance between RBC-NPs of EpCAM+ cells (MCF-7) and TT-RBC-NPs of EpCAM+ cells (MCF-7) (*p* < 0.05). ** the significance between TT-RBC-NPs of EpCAM– cells (Fibroblast) and TT-RBC-NPs of EpCAM+ cells (MCF-7) (*p* < 0.05).

Additionally, the binding capability of TT-RBC-NPs was further investigated using flow cytometer. As shown in Figure 8a, immunofluorescent analysis of the negative control group comprising untreated fibroblast and MCF-7, as expected, gave a weak signal. Also, the non-targeted RBC-NPs group treated both the fibroblast and MCF-7 gave a very weak signal. Similar to the results from the previous experiment, the MCF-7 targeted cells treated with TT-RBC-NPs gave a strong signal, verifying good TT-RBC-NPs targeting efficacy and uptake, while the fibroblast cells and non-targeted cells treated with TT-RBC-NPs gave a weak signal, indicating minimal binding and uptake of the TT-RBC-NPs. Notably, the binding capability determined the targeted TT-RBC-NPs had a 48-fold higher fluorescence intensity than the non-targeted RBC-NPs. Therefore, the flow cytometer imaging analysis (Figure 8b) demonstrated that the mean fluorescence intensity of the MCF-7 cells treated with the TT-RBC-NPs was significantly higher than the non-targeted control group, showing a noticeable targeting effect.

### 2.7. Nanoparticle Penetration and Uptake in Three-Dimensional (3D) Spheroids

**Figure 9:**
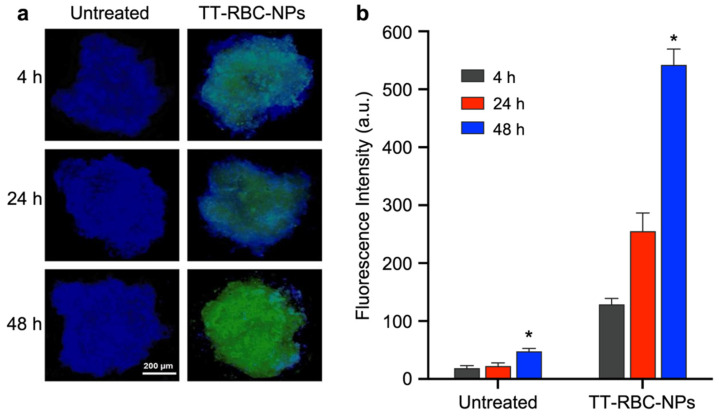
TT-RBC-NPs penetration in tumor spheroids. (**a**) Fluorescence images of MCF-7 (EpCAM+) spheroids with and without treated with TT-RBC-NPs (green channel). The nucleus was stained with DAPI (blue channel). Scale bar = 200 µm. (**b**) Quantification of mean fluorescence intensities of TT-RBC-NPs on MCF-7 spheroids with incubation times of 4, 24 and 48 h. Bars represent mean ± SD (*n* = 3). * the significance between Untreated and TT-RBC-NPs at 48 h incubation (*p* < 0.05).

The in vitro nanoparticle penetration of TT-RBC-NPs in the 3D spheroid model was next investigated. In order to confirm the nanoparticle penetration to the 3D tumor breast cancer spheroids, which overexpress EpCAM on the cell surface. The fluorescence images of MCF-7 (EpCAM+) spheroids with and without treatment by TT-RBC-NPs (green) for 4, 24 and 48 h were visualized (Figure 9a). The results indicate that the penetration of the TT-RBC-NPs in tumor spheroids increases over time, as the longer the incubation time, the higher the green signal detected inside the tumor spheroid. Additionally, the mean fluorescence intensities of the TT-RBC-NPs on MCF-7 spheroids with incubation times of 4, 24, and 48 h were quantified (Figure 9b). The results corresponded with the images, showing a higher green fluorescence intensity in the MCF-7 spheroids when treated with TT-RBC-NPs over time. Moreover, at 48 h incubation time, the TT-RBC-NPs penetrated inside the tumor spheroid resulted in approximately 2.9-fold higher than at 24 h incubation time. Overall, the results indicate that the TT-RBC-NPs have the potential to accurately target breast cancer cells and also enhance nanoparticle penetration and uptake properties.

### 2.8. Blood Compatibility of TT-RBC-NPs

**Figure 10:**
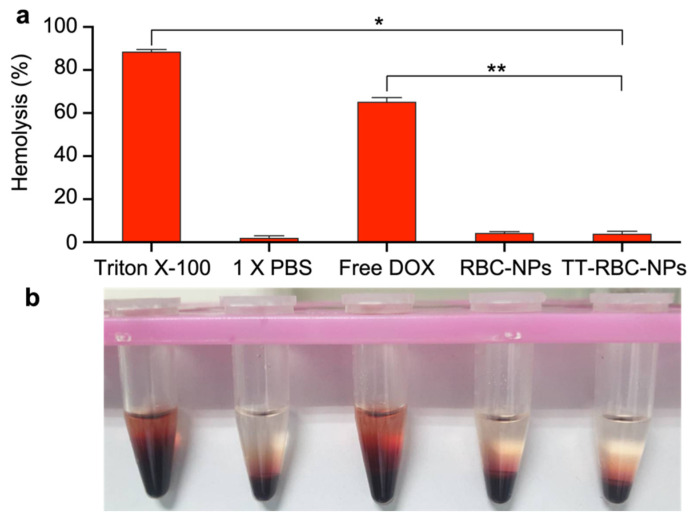
Hemotoxicity of TT-RBC-NPs. (**a**) Hemolysis of Triton X-100, PBS, free DOX, RBC-NPs and TT-RBC-NP (equivalent of 1 µg/mL DOX). Data represents mean ± SD (*n* = 3). (**b**) Images of red blood cells treated with Triton X-100, PBS, free DOX, RBC-NPs and TT-RBC-NP (equivalent of 1 µg/mL DOX) for 48 h. * the significance between Triton X-100 and TT-RBC-NPs at 48 h incubation (*p* < 0.05). ** the significance between Free DOX and TT-RBC-NPs at *48* h incubation (*p* < 0.05).

Nanoparticle hemolytic capabilities are widely utilized in the study of nanoparticle interactions with blood components. Here, the induced hemolysis was assessed by comparing the measured hemoglobin concentration to the total hemoglobin concentration [50]. To ensure that the TT-RBC-NPs had no harmful effects on the blood components, the hemolysis properties of the nanoparticles was studied. At the end of the incubation period (48 h), red blood cells were pelleted, and the supernatant analyzed for lysed hemoglobin (Figure 10a). The results verify that the TT-RBC-NPs did not cause hemolysis of the red blood cells. Conversely, Triton X-100 (positive control) resulted in RBC lysis (Figure 10b). Hypotonic treatment of RBC served as 100% hemolysis (data not shown). Free DOX caused approximately over 60% hemolysis, whereas RBC-NPs and TT-RBC-NPs resulted in only ~5% hemolysis. Similarly, PBS and the TT-RBC-NPs had a low level of hemolysis, suggesting the TT-RBC-NPs could potentially reduce side effects of DOX when encapsulated in biomimetic coated nanoparticles. According to the findings of a number of different investigations that were carried out in vitro, a proportion of hemolysis ranging from 5% to 25% is regarded as being “of no concern”. These nanoparticles are acceptable for potential biomedical use [51,52,53]. Furthermore, cellular uptake by macrophage cells of non-coated bare PLGA nanoparticles (Bare NPs), RBC-NPs, and TT-RBC-NPs compared with PEGylated nanoparticles (PEG-NPs), the current gold standard for long circulating nanoparticles, was performed (Figure S3). We found that both RBC-NPs and TT-RBC-NPs have significant low macrophage uptake compared with non-coated bare PLGA nanoparticles (bare NPs). Moreover, they have the same low uptake by macrophages as PEG-NPs, the current gold standard. This confirms that TT-RBC-NPs possess immune system evasion abilities.

## 3. Materials and Methods

### 3.1. Materials

Human packed red blood cells were donated by the Faculty of Medicine Blood bank, at Prince of Songkla University, Thailand. Carboxy-terminated 50:50 poly(d, l-lactide-co-glycolide) (PLGA) polymer 0.66 dL/g was purchased from LACTEL Absorbable Polymers (Birmingham, AL, USA). Doxorubicin hydrochloride was purchased from Pfizer Laboratories (New York, NY, USA). Phosphate buffer solution (PBS) was acquired from Thermo-Fisher (Waltham, MA, USA). Both the bicinchoninic acid (BCA) assay kit and paraformaldehyde were procured from Millipore Sigma (St. Louis, MO, USA). Streptavidin, Streptavidin-Fluorescein Isothiocyanate (streptavidin-FITC), biotinylated anti-EpCAM monoclonal antibody was bought from Biolegend (San Diego, CA, USA). The MTS Assay Kit (Cell Proliferation) (colorimetric) was purchased from Abcam (Cambridge, MA, USA). MCF7 breast cancer cells and PCS-201-010 dermal fibroblast were purchased from ATCC (Manassas, VA, USA). 1,2-distearoyl-*sn*-glycero-3-phosphoethanolamine-*N*-[biotin(polyethylene glycol)-2000] (ammonium salt) (DSPE–PEG-biotin) was purchased from Avanti Polar Lipids (Alabaster, AL, USA). LIVE/DEAD Cell Imaging Kit (488/570) was bought from Thermo-Fisher Scientific (Waltham, MA, USA). CellTiter-Glo^®^ was purchased from Promega (Madison, WI, USA). Other chemicals and reagents were from Millipore Sigma (St. Louis, MO, USA), or Thermo-Fisher (Waltham, MA, USA).

### 3.2. Preparation of Nanoparticles

The doxorubicin (DOX) loaded in PLGA cores (DOX-NPs) were prepared by a double emulsion process. Briefly, DOX was dispersed in 25 μL of 500 mM Tris-HCl at pH 8 as the inner phase, and then sonicated at 70% pulsed power (2 seconds on/1 second off) for 2 min with 500 μL of PLGA in dichloromethane (DCM) at 10 mg/mL. The resulting solution was then added to 5 mL of 10 mM Tris-HCl at pH 8 and further sonicated for 2 min. The obtained solution was added to 10 mL of 10 mM Tris-HCl at pH 8 and stirred gently for 4 h to assist evaporation in a fume hood. Red blood cell (RBC) membrane extraction was prepared according to our previously reported procedure with some modifications [54]. Human RBCs were washed with 1× PBS before hypotonic treatment with 0.25× PBS and centrifuged at 800× *g* for 5 min to remove the hemoglobin. The resulting RBC membrane pellets were collected, and reconstituted in DI water.

RBC membrane vesicles were first functionalized with anti-EpCAM antibodies prior to coating onto PLGA cores. Membrane functionalization was performed using the lipid-insertion method [55]. First, biotinylated RBC membrane (RBC–biotin) was prepared by suspending 500 μL of RBC membrane vesicles in 5 mL 1× PBS (pH 7.4) and then incubated with 10 μg DSPE–PEG–biotin (3700 D, 100 μg/mL) at a temperature of 37 °C for 30 min and stirred continuously. The RBC–biotin was then washed twice with 1× PBS and centrifuged at 400× *g* for 5 min and then resuspended in 5 mL 1× PBS. Then 1 µg of streptavidin-FITC and streptavidin (1: 1 streptavidin wt. ratio) solution were incubated with RBC–biotin for 1 h at 4 °C. After which the solution was washed twice with PBS and centrifuged at 400× *g* for 5 min to separate any unbound molecules. Finally biotinylated anti-EpCAM antibodies were attached to the RBC membrane via streptavidin-biotin conjugation with 1 h incubation at a temperature of 4 °C and stirred continuously. Then any unbound molecules were removed by washing twice with PBS, and the final functionalized RBC membrane were collected. Modified membrane vesicles were produced by bath sonication for 2 min. The TT-RBC-NPs were fabricated by fusing functionalized RBC vesicles with DOX-loaded PLGA cores at desired membrane protein-to-core ratio using a previously established protocol [56]. FITC-modified RBC membrane without anti-EpCAM antibodies was coated onto DOX-loaded PLGA cores, herein denoted as RBC-NPs, and were prepared as non-targeted NPs control groups.

In brief, the various nanoformulations were synthesized. DOX-NPs is non-coated DOX loaded in PLGA cores, prepared by a double emulsion process. RBC-NPs is FITC-modified RBC membrane without anti-EpCAM antibodies coated onto DOX-loaded PLGA cores. In addition, the TT-RBC-NPs were FITC-modified RBC membrane with anti-EpCAM antibodies coated onto DOX-loaded PLGA cores.

### 3.3. Characterization of Nanoaparticles

The size and zeta potential of non-coated DOX-loaded NPs (DOX-NPs), RBC-NPs, and TT-RBC-NPs were obtained from dynamic light scattering (DLS) measurement using a Malvern ZEN 3600 Zetasizer (Malvern Panalytical, Malvern, Worcestershire, UK). The TT-RBC-NP morphology was characterized by a JEOL JEM-2010 Transmission electron microscopy (TEM, JEOL Ltd, Akishima, Tokyo, Japan). By placing 1 mg/mL of TT-RBC-NP onto a TEM glow-discharged carbon-coated grid, and then washed for 5 min with 10 droplets of distilled water and then stained 1 wt.% uranyl acetate. After which, the grid was dried and imaged by TEM set at 200 kV.

To evaluate the stability of the TT-RBC-NPs in a physiological buffer, 2 mg/mL of the nanoparticles were mixed with 2× PBS to achieve the final particle concentration of 1 mg/mL and 1× PBS. Likewise, the stability of the TT-RBC-NPs in fetal bovine serum (FBS) was evaluated by mixing nanoparticle suspensions in 1× PBS with 100% Fetal bovine serum (Hyclone) (final particle concentration of 1 mg/mL). The TT-RBC-NPs were stored at 4 °C over 1 month period. At predetermined time points, the TT-RBC-NPs were characterized for size and polydispersity index (PDI) using dynamic light scattering (DLS) measurements (Malvern ZEN 3600 Zetasizer).

To evaluate the loading yield and the encapsulation efficiency of the TT-RBC-NPs with varied initial drug inputs, DOX was evaluated by measuring its fluorescence (excitation at 480 nm; emission at 580 nm). A cumulative drug release study was performed by dialyzing samples against 1× PBS using Slide-A-Lyzer MINI Dialysis Cups (Sigma-Aldrich, St. Louis, MO, USA), with 3.5 kDa molecular weight cut-off. The dialysis cups were put into an enclosed beaker containing 2000 mL of 1× PBS buffer (pH = 7.4) release medium, then stirred at 200 rpm at 37 °C. At specific time points, samples were drawn from the buffered medium solution and substituted with the same volume of fresh buffer medium to preserve the constant pH and sink condition. DOX in NPs were extracted using 0.1 M HCl in acetonitrile solution. DOX concentration was evaluated by measuring its fluorescence (excitation at 480 nm; emission at 580 nm). All experiments were performed in triplicate and the results are shown as a mean of all three values (mean ± SD; *n* = 3).

### 3.4. In Vitro Therapeutic Efficacy on 2-Dimensional (2D) Cell Culture

To investigate the anti-cancer efficacy of the TT-RBC-NPs, in vitro cytotoxicity was performed using an MTS assay (Supplementary Materials). The MCF-7 were cultured in Dulbecco’s Modified Eagle Medium (DMEM) (Gibco-BRL) and supplemented with 10% fetal bovine albumin, 1% penicillin/streptomycin (Gibco-BRL), and l-glutamine (Gibco-BRL). All the cells were cultured to 60–80% confluency in culture flasks in a humidified incubator at 5% CO_2_ at 37 °C. 5000 cells/well were plated in 96-well microtiter plates at a final volume of 200 µL/well for 24 h before treatment. MCF-7 cells were treated with free DOX and TT-RBC-NPs at various DOX concentrations for 24 h. With 1× PBS as a negative control and 5% DMSO as a positive control. All the wells were then washed, and incubated in fresh culture medium for 72 h in a final volume of 200 µL/well, and an MTS viability assay was used to quantify the cell viability following the manufacturer’s protocol. Briefly, 20 µL of MTS reagent was added to each well and incubated for 2 h at 37 °C and 5% CO_2_. The plates were then gently shaken for 1 min on an orbital shaker, and absorbance was measured using a multiplate reader (Tecan, Infinite 200 PRO, Tecan Group Ltd., Männedorf, Switzerland) at OD = 490 nm. Next, the in vitro efficacy of free DOX, RBC-NPs, and TT-RBC-NP (equivalent to 1 µg/mL DOX) against MCF-7 cells was performed. In this assay, samples were incubated for 2 h, and the cells were subsequently washed and incubated in fresh medium for an additional 0, 24, 48, and 72 h before quantifying the toxicity using the MTS assay.

### 3.5. Three-Dimensional (3D) In Vitro Live/Dead Cell Imaging and Cellular Cytotoxicity Assay

Two-color fluorescence LIVE/DEAD Cell Imaging Kit (488/570) (ThermoFisher Scientific, R37601, Eugene, OR, USA) was used to visualize the cell viability of MCF-7 spheroids after TT-RBC-NP treatment. The MCF-7 cells were cultured in DMEM and supplemented with 10% fetal bovine albumin, l-glutamine, and 1% penicillin/streptomycin. MCF-7 spheroids were prepared as previously described [57]. Briefly, MCF-7 cells were cultured to 60–80% confluency at 5% CO_2_ in a humidified incubator at a temperature of 37 °C. Then 5000 cells/well of MCF-7 were plated in 96-well ultra-low attachment multiple well plates at a final volume of 200 µL/well for 72 h before treatment. The spherical aggregates produced from the MCF-7 cells (one spheroid/well) were then treated with free DOX, RBC-NPs, and TT-RBC-NPs at equivalent DOX concentrations of 1 µg/mL for 4, 24, and 48 h. The untreated group was used as a negative control, and all experiments were completed in triplicate. The spheroids were then washed with PBS. Then a two-color fluorescence LIVE/DEAD Cell Imaging Kit was prepared following the manufacturer’s protocol. Live Green (Comp. A) was added to Dead Red (Comp. B) and mixed to create a 2× working solution. Next, 50 µL of 2× working solution was added to each well and incubated for 15 min at 25 °C. Then the cells were imaged using LionHeart live cell imaging. Afterward, a CellTiter-Glo^®^ cell viability assay was performed to quantify cell viability on the spheroids treated with free DOX, RBC-NPs, and TT-RBC-NPs at equivalent DOX concentrations of 1 µg/mL for 4, 24, and 48 h. According to the manufacturer’s protocol, the medium was removed, and 100 µL of CellTiter-Glo^®^ reagent was added in each well, mixed for 2 min, followed by incubation for 30 min at room temperature. The luminescence was measured using a plate reader (Tecan Infinite 200 PRO, Männedorf, Switzerland).

### 3.6. In Vitro Targeting Ability of TT-RBC-NPs

To evaluate the TT-RBC-NPs’ targeting ability on the targeted cells (EpCAM+ MCF-7 breast cancer cells), non-targeted cells (EpCAM− skin fibroblast cells) were used as a control [58,59,60,61]. The targeting capability was assessed using both fluorescent microscopy and flow cytometer. For the fluorescent imaging study, 5000 cells/well of MCF-7 and skin fibroblasts were plated in 96-well tissue culture plates at a final volume of 200 µL/well until 60–80% confluency was reached in a humidified incubator at 37 °C, 5% CO_2_. On the day of the experiment, the cells were washed with pre-warmed 1× PBS and incubated in a pre-warmed DMEM medium supplemented with 10% fetal bovine albumin, 1% penicillin/streptomycin, and L-glutamine. Next, RBC-NPs and TT-RBC-NPs were added to the cells at 1 mg/mL final concentration. After 1 h of incubation at 37 °C, the cells were washed with 1× PBS three times, fixed with 4% paraformaldehyde in PBS for 15 min at room temperature, and stained with 4′,6-diamidino-2-phenylindole (DAPI). Finally, the cells were washed with 1× PBS to remove unbound staining and then imaged using a deconvolution scanning fluorescence microscope (Lionheart FX Automated Microscope, BioTek Instrument, Santa Clara, CA, USA). Digital images of blue (4′,6-diamidino-2-phenylindole; DAPI), green (FITC) and the images overlaid and deconvoluted using Gen5 software (Biotek Instruments Inc., Winooski, VT, USA). All images were processed for signal intensity by CellProfiler image analysis software (version 3.1.8, Cimini Lab, Cambridge, MA, USA). The qualification was achieved with 10× images (*n* = 3).

For the flow cytometer study, EpCAM+ MCF-7 breast cancer cells and EpCAM− skin fibroblast cells were cultured in Dulbecco’s Modified Eagle Medium (DMEM) (Gibco-BRL) and supplemented with 10% fetal bovine albumin, 1% penicillin/streptomycin (Gibco-BRL), and l-glutamine (Gibco-BRL). Cells were grown to 60–80% confluency, and then detached from the culture flasks using 0.25% Trypsin-EDTA solution (Gibco-BRL), and the cells were collected by centrifugation and washed twice with 1× PBS at 4 °C. One million cells/mL were prepared for optimal flow cytometer performance. First, the RBC-NPs and TT-RBC-NPs were added to the relevant cells and incubated in the dark for 1 h at 4 °C. This was followed by washing twice with 2 mL PBS to remove unbound nanoparticles, then resuspended with fresh medium prior to flow cytometer analysis.

### 3.7. Nanoparticle Penetration in Three-Dimensional (3D) Spheroids

A 3D spheroid model was used to observe the penetration of the TT-RBC-NPs in tumor spheroids. The MCF-7 spherical aggregates were prepared as previously described. The spheroids (one spheroid/well) were then treated with TT-RBC-NPs equivalent to 1 µg/mL DOX concentration, for 4, 24, and 48 h. The untreated group was used as a negative control. The wells were washed with 1× PBS and then fixed in 4% paraformaldehyde in PBS for 1 h. The fixed spheroids were washed and stained with 4′,6-diamidino-2-phenylindole (DAPI), and the treated spheroids were then imaged. The TT-RBC-NPs’ fluorescence intensities on the spheroids were quantified by LionHeart live cell imaging.

### 3.8. Blood Compatibility

In vitro hemolytic properties of the nanoparticles were evaluated with some modifications from the Nanotechnology Characterization Laboratory (NCL)’s published protocol [50]. Briefly, human blood was collected in the presence of ethylenediaminetetraacetic acid (EDTA), and the serum was removed. Then the red blood cells were suspended at 8 × 10^9^ cells/mL and exposed to treatment groups including 0.25 mg/mL Triton X-100, PBS, free DOX, RBC-NPs, and TT-RBC-NP (equivalent of 1 µg/mL DOX) for 48 h at 37 °C. Following centrifugation, the absorbance of hemoglobin in the supernatants was measured and used to quantify the hemolysis percentage. Triton-X at 0.25 mg/mL was used as the positive control and 1× PBS used as the negative control.

### 3.9. Statistical Analysis

All experiments were accomplished in triplicate. Results and experimental data represent mean ± SD. All statistical analyses used GraphPad Prism 6.0 software (GraphPad Software Inc., San Diego, CA, USA). Pairwise statistical comparisons were performed using the unpaired, two-tailed Student’s *t*-test. Furthermore, one-way ANOVA was used to compare the means of two or more independent groups to assess whether the means were statistically different, with *p* < 0.05 being considered statistically significant.

## 4. Conclusions

The application of RBC membranes as a stealth shield for drug delivery systems against the immune system has attracted increasing interest in the application of bio-materials with low immunogenicity, high biocompatibility, and prolonged circulation times. Applying a RBC cell membrane coating to nanoparticles facilitates the translocation of the key features and functionality of the membrane cells to the nanoparticle core. Previously, an in vivo study showed that murine RBC-cloaked nanoparticles were not only efficacious against a murine lymphoma model but also safe and immunocompatible [29] This study aimed to translate RBC-cloaked NPs for clinical applications using human RBC NPs with enhanced targeting functionalization. Furthermore, demonstrating that the TT-RBC-NPs had excellent targeting ability to EpCAM overexpressed breast cancer cells over non-targeted cells, which resulted in higher penetration and cellular uptake. In particular, the TT-RBC-NPs demonstrated improved cytotoxic efficacy over non-targeted RBC NPs and free drug in both 2D and 3D in vitro breast cancer models. Furthermore, the TT-RBC-NPs specifically targeted MCF-7 breast cancer cells with minimum binding to non-targeted cells and showed good penetration inside the 3D tumor spheroid. Moreover, the PLGA core provides good degradation characteristics and biocompatibility and is recognized as being a safe material for biomedical applications.

Our future research objective is to further optimize this nanotheranostic for clinical translation: With the current design, the imaging agent can be simply replaced with other probes such as cyanine-based fluorescent dyes, IRDye800CW, NIR fluorophores, MRI contrast agents (T_1_ and T_2_ agents), or radiopharmaceuticals. It should be noted, though, that several aspects such as multifunctional targeting agents, nanoparticle-mediated combination therapy, large-scale synthesis, and clinical safety profiles remain to be investigated before translating this platform to human use. This targeted biomimetic drug delivery platform has the potential to be further developed in the future for novel personalized cancer treatment.

## Data Availability

The data presented in this work are contained within the article.

## Supplementary Materials

The following supporting information can be downloaded at: https://www.mdpi.com/article/10.3390/molecules27196473/s1, Figure S1: Drug release profiles and kinetics of TT-RBC-NPs. (a) Doxorubicin release profiles of TT-RBC-NPs. (b) the drug-release percentage was plotted against the square root of time, which yielded linear fittings using a diffusion-dominant Higuchi model; Figure S2. In vitro cytotoxicity of non-loaded bare PLGA NPs, non-loaded RBC-NPs and non-loaded TT-RBC-NPs at 250 μg/mL of PLGA when incubated with MCF-7 breast cancer cells for 24 hours. With 1×PBS as an untreated negative control. All the wells were washed, and incubated in fresh culture media for 72 hours before tested with MTS viability assay; Figure S3. Macrophage Uptake. Fluorescence analysis of cellular uptake by macrophage cells of non-coated bare PLGA nanoparticles (Bare NPs), PEG-NPs, RBC-NPs, and TT-RBC-NPs.

## Sample Availability

Samples of the compounds are not available from the authors.
