# Supplementary material for: Biomimetic Targeted Theranostic Nanoparticles for Breast Cancer Treatment
Source: Molecules. 2022 Oct 1;27(19):6473. doi: 10.3390/molecules27196473 (PMC9571674; doi:10.3390/molecules27196473)
Supplement: Supplementary file 1 [file molecules-27-06473-s001.zip › molecules-1863132-supplementary.pdf]

molecules

MDPI

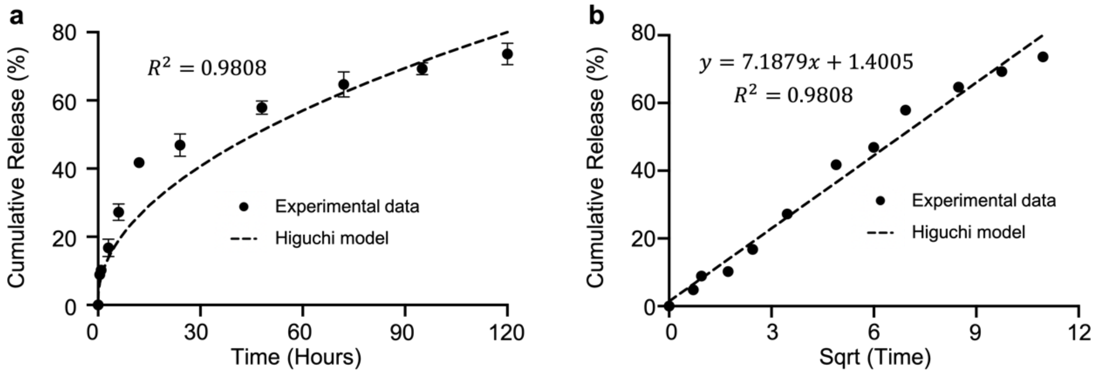


**Figure S1. Drug release profiles and kinetics of TT-RBC-NPs.** (a) Doxorubicin release profiles of TT-RBC-NPs. (b) the drug-release percentage was plotted against the square root of time, which yielded linear fittings using a diffusion-dominant Higuchi model.

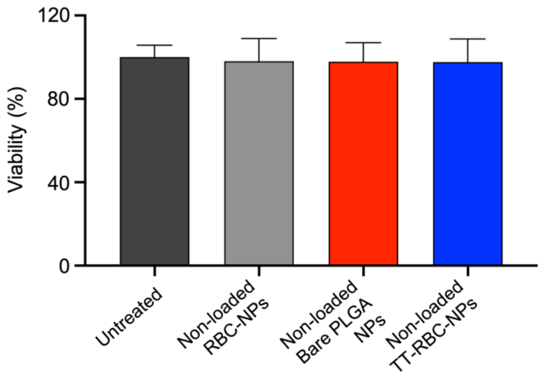


**Figure S2.** ***In vitro*** **cytotoxicity** of non-loaded bare PLGA NPs, non-loaded RBC-NPs and non-loaded TT-RBC-NPs at 250 μg/mL of PLGA when incubated with MCF-7 breast cancer cells for 24 hours. With 1× PBS as an untreated negative control. All the wells were washed, and incubated in fresh culture media for 72 hours before tested with MTS viability assay.

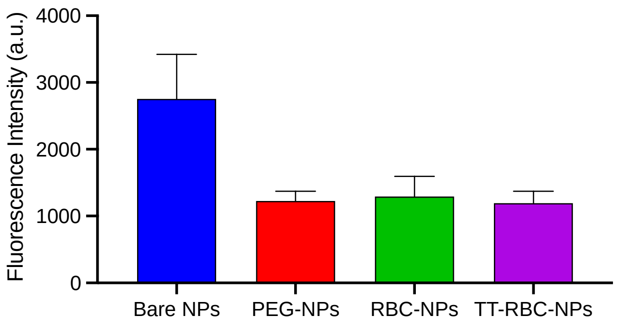


**Figure S3. Macrophage Uptake.** Fluorescence analysis of cellular uptake by macrophage cells of non-coated bare PLGA nanoparticles (Bare NPs), PEG-NPs, RBC-NPs, and TT-RBC-NPs.
